# TEACHING ABOLITIONIST GERONTOLOGY IN INTERDISCIPLINARY LEARNING SPACES

**DOI:** 10.1093/geroni/igad104.2027

**Published:** 2023-12-21

**Authors:** Ian Johnson

**Affiliations:** University of Texas- San Antonio, San Antonio, Texas, United States

## Abstract

The discourse surrounding population aging has now infiltrated prisons, jails, and the ‘institutional circuit’ of homeless services, mental health systems, substance abuse treatment, and safety-net healthcare. Students of gerontology must be prepared to consider the experiences of people most affected by the criminal-legal apparatus. Conversely, those training to be social service and healthcare professionals within and in opposition to these systems must be prepared to. Through a conceptual review of the literature and experiential knowledge gathered through curriculum design and implementation, this presentation will outline a pedagogical framework for understanding the effects of transcarceration across the life course and in later life. This framework will be presented in conjunction with tools and strategies for guiding and enhancing interdisciplinary dialogue about the intersections of eldercare and punishment. This presentation will prompt discussion on the responsibilities of aging care providers and gerontological researchers in realizing an abolitionist future.

